# Prevalence of anti-phospholipase A2 receptor antibodies in Japanese patients with membranous nephropathy

**DOI:** 10.1007/s10157-014-1054-2

**Published:** 2014-11-21

**Authors:** Shin’ichi Akiyama, Mari Akiyama, Enyu Imai, Takenori Ozaki, Seiichi Matsuo, Shoichi Maruyama

**Affiliations:** 1 Division of Nephrology, Department of Internal Medicine, Nagoya University Graduate School of Medicine, 65 Tsurumai-cho, Showa-ku, Nagoya, 466-8550 Japan; 2 Nakayamadera Imai Clinic, 2 Nakayamadera, Takarazuka, 665-0861 Japan

**Keywords:** Phospholipase A2 receptor, Antibody, Membranous nephropathy, Prevalence, Japan, Western blot

## Abstract

**Background:**

Membranous nephropathy (MN) is the leading cause of nephrotic syndrome in adults. Anti-M-type phospholipase A2 receptor (anti-PLA2R) antibodies are found in most patients with idiopathic MN (iMN) worldwide, but the prevalence of anti-PLA2R antibodies among Japanese patients with MN is unknown. In this study, we determined the prevalence of anti-PLA2R antibodies in Japanese patients with MN.

**Methods:**

The study population of our retrospective cross-sectional consisted of 131 patients with biopsy-proven MN who had not received any immunosuppressive treatments at time of both renal biopsy and serum sample collection. Of these, 100 had iMN and 31 had secondary MN (sMN). The circulating anti-PLA2R antibodies were analyzed using a highly sensitive Western blot analysis. Analysis was performed under non-reducing conditions with a human glomerular extract at serum dilutions of 1:25, 1:10, and 1 as the primary antibody.

**Results:**

Anti-PLA2R antibodies were detected in 53 (53 %) of 100 patients with iMN and 0 (0 %) of 31 patients with sMN. The prevalence of anti-PLA2R antibodies was higher in patients with nephrotic syndrome (61 %) than in patients without nephrotic syndrome (43 %). The number of patients with serum albumin ≤3.0 g/dL was significantly higher in those with anti-PLA2R antibodies (92 %) than that in those without them (68 %).

**Conclusions:**

Anti-PLA2R antibodies were found in Japanese patients with iMN; however, the prevalence was lower than that of any other Asian country. This may indicate that the presence of other pathogenic antigens plays a significant role in Japanese patients with iMN.

## Introduction

Membranous nephropathy (MN) is a leading cause of nephrotic syndrome in adults. It is classified as either idiopathic (iMN) or secondary (sMN) depending on its etiology. Overall, 40 % of Japanese patients with nephrotic syndrome and 33–50 % of patients with nephrotic syndrome in other countries either develop end-stage renal disease within 20 years of onset. Mortality from MN is high due to complications, such as infection, cardiovascular events, or malignancy [1–6]. The pathogenesis of iMN was not understood until the landmark study by Beck et al. in 2009, which demonstrated that the major target antigen of autoantibodies in iMN is an M-type phospholipase A2 receptor (PLA2R) expressed on podocytes [7].

The anti-PLA2R antibody specifically and accurately recognizes a 3-dimensional epitope supported by intramolecular disulfide bonds in the PLA2R protein. *N*-glycosylation of the PLA2R protein is not necessary for the interaction between the anti-PLA2R antibody and the PLA2R protein [7]. The major subclass of anti-PLA2R antibody in serum samples from patients with iMN is immunoglobulin G4 (IgG4). Hofstra et al. [8] reported that levels of circulating anti-PLA2R antibodies correlate strongly with the level of proteinuria. Beck et al. [9] reported that changes in the level of anti-PLA2R antibodies are associated with the level of proteinuria and precede corresponding changes in disease activity. From these observations, it would be expected that the anti-PLA2R antibody can be used as a biomarker in the differential diagnosis of iMN, monitoring of disease activity, and prediction of disease outcome. Moreover, Hoxha et al. [10] reported that the level of expression of PLA2R on the cell surface of podocytes of iMN patients with anti-PLA2R antibodies was higher than in iMN patients without the antibodies. Stanescu et al. [11] showed that polymorphisms of the PLA2R and HLA-DQA1 alleles are associated with the risk of developing iMN. They concluded that the HLA-DQA1 allele on chromosome 6p21 is most closely associated with iMN in people of white ancestry.

Beck et al. [7] also revealed that the anti-PLA2R antibody was found in 26 of 37 patients with iMN, but was not found in patients with sMN, disease controls, or healthy controls. Its prevalence in American patients with iMN was 70 %. The overall prevalence of anti-PLA2R antibodies in iMN patients with nephrotic syndrome is reported to be 66–98 % [7, 8, 12–14]. The wide range may be attributable to genetic and regional differences or to differences in the detection methods employed. The prevalence of anti-PLA2R antibodies in Japanese patients with MN has not yet been determined. Western blot analysis with chemical luminescence is the prevailing method for detecting anti-PLA2R antibodies in serum, but there are some technical problems with this method. In this study, we determined the presence of anti-PLA2R antibodies in Japanese patients with untreated MN using a highly sensitive method of Western blot analysis we developed.

## Materials and methods

### Patients

A total of 106 patients with iMN and 35 patients with sMN admitted to a hospital between 2005 and 2011 were enrolled in this study. Patients were diagnosed with iMN or sMN based on both a renal biopsy and screening for clinical or laboratory signs of a cause for sMN. Six patients with iMN and 4 patients with sMN were excluded from this study because they were treated with immunosuppressive therapy at the time of biopsy. Blood samples and timed urine samples were collected from a total of 100 iMN patients and 31 sMN patients for the measurement of serum albumin, creatinine, total protein total cholesterol, IgG, anti-PLA2R, and urinary protein. Patients with sMN (*n* = 31) included 11 patients with immune-associated disease, including Castleman’s disease (*n* = 1) and SLE (*n* = 10); 6 patients with neoplasia, including lung cancer (*n* = 4), urinary bladder cancer (*n* = 1), and other cancer (*n* = 1); 6 patients whose disease was associated with the use of the anti-rheumatoid drug bucillamine; 2 patients with hepatitis B virus infection; and 6 patients with other primary diseases. The study protocol was approved by the ethics committees of our institution (#1135-14) and was conducted in accordance with the ethical principles stated by the Declaration of Helsinki.

### Antigen

We used human glomerular extract (HGE) as a source of native glomerular PLA2R for detection of circulating anti-PLA2R antibodies in patient serum. A human kidney was obtained from a patient with renal cancer who underwent surgery at out institution. Glomeruli were collected from healthy portions of the kidney using a series of graded sieves, were rinsed with cooled phosphate buffer saline, and were dissolved with RIPA buffer with proteinase inhibitor cocktail (Roche). Insoluble debris was removed by centrifugation. To eliminate contamination with donor endogenous IgG, the HGE was treated with HiTrap Protein G HP column (GE). The prepared HGE was quality checked using Western blot to ensure there were no reactive IgG against anti-human IgG murine antibody, and a reactive PLA2R protein against both anti-human PLA2R rabbit polyclonal antibody (Atlas antibodies, product number: HPA012657) and a positive control serum containing anti-PLA2R in HGE. All reagents were obtained from Sigma Japan or Wako Pure Chemicals if not specified otherwise.

### Immune reaction in Western blot analysis

The HGE was heated with 4x LDS-sample buffer (Invitrogen) at 70 °C for 10 min and then electrophoresed under non-reducing conditions on the NuPAGE 4–12 % polyacrylamide Tris-Bis gel (Invitrogen) with MOPS SDS running buffer (Invitrogen). The proteins were separated by SDS-PAGE and transferred to a PVDF membrane. The membrane was blocked with Blocking One (Nacalai tesque) at 25 °C for 60 min. In the first immune reaction, all patient serum samples were diluted with a diluent, which was mixed with 80 % PBST (phosphate buffer saline:Tween 20 = 99.8:0.2) and 20 % of Blocking One, at a dilution of 1:25 and reacted with the blocked membrane at 37 °C for 60 min. For samples with negative results at a dilution of 1:25, we retested all samples at a dilution of 1:10 or 1 and confirmed non-reactivity based on the negative results at the higher concentration of serum. In the second immune reaction, a horseradish peroxidase (HRP) conjugated mouse monoclonal antibody against human IgG Fc (Abcam, product number: ab99759) was diluted at a dilution of 1:7500 and reacted with a membrane at 37 °C for 60 min.

### Signal detection in Western blot analysis

To obtain a high sensitivity and resolution, we attempted to pretest a signal detection method of the bound secondary antibody. In the pretest, Western blot analysis was conducted with HGE as antigen, serial diluted anti-PLA2R human antibodies known to be the same concentration as the first antibody, and the anti-human IgG Fc mouse monoclonal antibody as the secondary antibody. The serial diluted anti-PLA2R human antibodies were prepared from attached reagents as standard anti-PLA2R human antibody for concentration calibration in commercial ELISA kit (Euroimmun AG). After reacting with the secondary antibody, we weighed the chromogenic method against the chemical luminescence method. The bound secondary antibody was detected by chemical luminescence with ImmunoStar LD or chromogenic reaction with a 3, 3′, 5, 5′-tetramethylbenzidine (TMB) as a substrate of HRP. Images of reacted bands on the PVDF membrane were acquired by a CCD imager (LAS4000, GE) in the Chemical luminescence or digital camera (Canon) using the chromogenic method. We defined that the serum sample was positive for anti-PLA2R if a single reactive band appeared in the position of native PLA2R protein (approximately 180 kDa) on the PVDF membrane. The acquired figures were prepared using Photoshop CS5 (Adobe systems). In this study, all patient serum used to estimate the prevalence of anti-PLA2R antibody was analyzed using the chromogenic reaction method.

### Statistical analysis

For data description, continuous variables with symmetric distribution were presented as the mean ± SD, and non-normally distributed variables were expressed as medians (25–75 % interquartile range). *T* tests were used for normally distributed data, and the Mann–Whitney *U* rank test was used for non-parametric data. The Dunn method was performed for multiple comparisons in non-parametric analysis. Categorical variables were described as number and percentages, and the data were analyzed with *χ* ^2^ tests. The differences were considered significant with a *P* value <0.05. All statistical analyses were performed with JMP version 11.0.0 (SAS Institute, USA) for Mac.

## Results

### Western blot analysis for the detection of anti-PLA2R antibodies

**Fig. 1:**
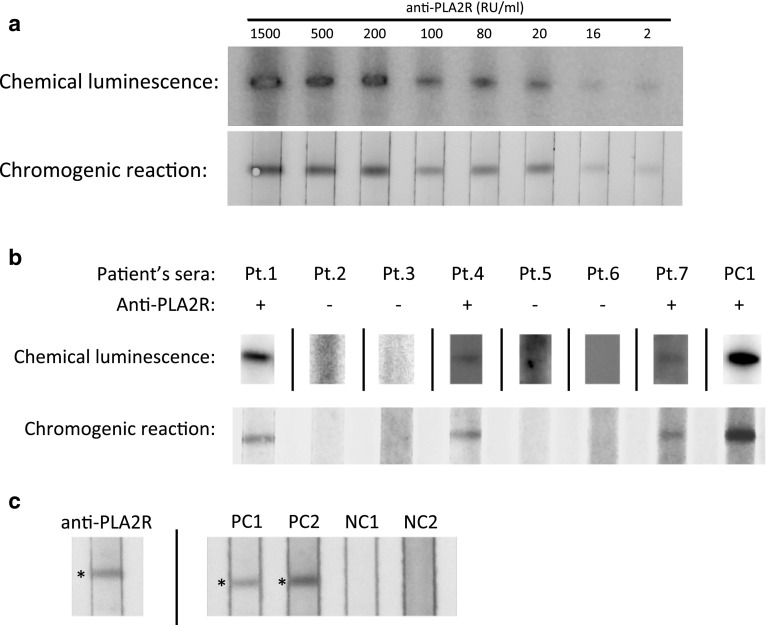
**a** The results of comparative investigation between chemical luminescence and chromogenic reaction in Western blot analysis with serial concentration of human anti-PLA2R antibody. The chemical luminescence showed blurred reactive bands in all concentrations and bleached bands with high background in high concentrations of anti-PLA2R antibodies. The chromogenic reaction showed sharply defined bands with low background in all range levels of anti-PLA2R antibodies. **b** The chromogenic reaction visualized higher contrast reactive band images compared to that of chemical luminescence in Western blot analysis with HGE and serum from some of the patients with iMN who were enrolled in this study. **c** The left image shows the existence of native PLA2R protein in HGE demonstrated using commercial anti-PLA2R rabbit polyclonal antibody. The right image shows that the positive control serum reacted with native PLA2R protein in HGE, whereas the negative control serum did not. *Pt* patient, *PC* positive control, *NC* negative control

Figure 1a shows blurred reactive bands with high-background on chemical luminescence at all levels of anti-PLA2R antibodies and all exposure times. Bleached bands appeared in the lane with a high concentration of anti-PLA2R antibodies. The chromogenic reaction method showed sharply defined bands with low-background in the wide-range levels (2–1500 RU/mL) of anti-PLA2R antibody. According to an instruction manual of a commercial anti-PLA2R antibody measurement kit from Euroimmun AG, the 16 RU/mL of anti-PLA2R antibody corresponds to the threshold value for a positive determination. Timmermans et al. [15] suggested that 2 RU/mL should be the recommended cutoff. Figure 1b shows the results of a comparison test using serum from some of the patients with iMN. In chemical luminescence, most were weak bands covered by high-background signal. Therefore, we selected the chromogenic reaction as the detection method for secondary antibody in this study.

Figure 1c shows the reactive bands of a commercial anti-PLA2R rabbit polyclonal antibody and positive and negative control serum from patients with iMN in an American cohort against PLA2R in HGE. We confirmed the existence of native PLA2R in HGE based on the fact that all reactive bands were represented at approximately 180 kDa on the PVDF membrane.

### Prevalence of anti-PLA2R in Japanese patients with membranous nephropathy

**Fig. 2:**
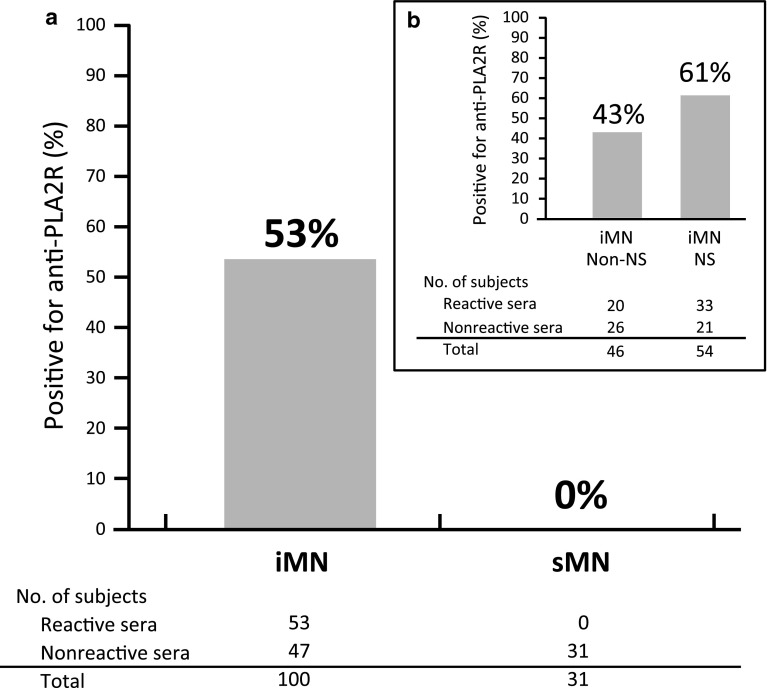
The prevalence of circulating anti-PLA2R antibody in serum from Japanese patients with membranous nephropathy. **a** The anti-PLA2R antibodies are specifically detected in 53 % of patients with iMN and no patients with sMN. **b** The anti-PLA2R antibodies were positive in 61 % of the iMN patients with nephrotic syndrome (UP ≥3.5 g/day and serum albumin ≤3.0 g/dL) and in 43 % of iMN patients without nephrotic syndrome. *NS* nephrotic syndrome

Table 1 shows the clinical characteristics of the patients with iMN and sMN at the time of kidney biopsy. The mean age was 67 in iMN patients and 61 in sMN patients. We found that 53 % (53 of 100) of iMN patients were positive for anti-PLA2R antibody whereas no patients with sMN (0 of 31) were positive (Fig. 2). The 53 anti-PLA2R antibody positive serum samples from iMN patients consisted of 43, 3, and 7 anti-PLA2R positive serum samples that were found at dilution levels of 1:25, 1:10 and, 1, respectively. From these results, we concluded that 43 patients had a high titer, 3 had a middle titer, and 7 had a low titer of anti-PLA2R antibodies. Anti-PLA2R antibodies were positive in 61 % (33 of 54) of the iMN patients with nephrotic syndrome (UP ≥3.5 g/day and serum albumin ≤3.0 g/dL) and in 43 % (20 of 46) of iMN without nephrotic syndrome.

**Table 1:** Characteristics of patients with idiopathic and secondary membranous nephropathy

	IMN (*n* = 100)	SMN (*n* = 31)
Male, *n* (%)	63 (63)	20 (65)
Age at diagnosis (years)	67 ± 9	61 ± 12
Urinary protein (g/day)	4.1 (2.6–6.4)	4.9 (1.8–6.6)
Urinary protein ≥3.5 g/day, *n* (%)	59 (59)	19 (61)
Serum albumin (g/dL)	2.4 ± 0.7	2.5 ± 0.9
Serum albumin ≤3 g/dL, *n* (%)	81 (81)	21 (68)
Both urinary protein ≥3.5 g/day and serum albumin ≤3 g/dL, *n* (%)	54 (54)	17 (55)
Serum total protein (g/dL)	5.2 ± 0.8	5.8 ± 1.1
Serum creatinine (μM)	72.1 (61.9–88.4)	64.5 (54.8–84.0)
eGFR (ml/min/1.73 m^2^)	65.5 (54.3–77.8)	72.0 (58.0–97.0)
Serum IgG (mg/dL)	727 (563–1038)	1182 (767–1721)
Total cholesterol (mM)	7.6 (6.2–9.4)	6.1 (5.1–8.5)
Recognized underlying membranous nephropathy inducible disease		
Immune-associated disease [*n* (%)]	0 (0)	11 (35.5)
Neoplasia [*n* (%)]	0 (0)	6 (19.4)
Drugs [*n* (%)]	0 (0)	6 (19.4)
Infections [*n* (%)]	0 (0)	2 (6.4)
Other [*n* (%)]	0 (0)	6 (19.4)

The data are expressed as the number (%), mean ± SD or median (interquartile range)

*iMN* idiopathic membranous nephropathy, *sMN* secondary membranous nephropathy, *eGFR* estimated glomerular filtration rate

### Relationship between patient characteristics and the anti-PLA2R antibodies in patients with iMN

We examined the patient characteristics of iMN patients negative for anti-PLA2R antibodies and positive for anti-PLA2R antibodies (Table 2). The number of patients with serum albumin ≤3.0 g/dL was significantly higher in iMN patients with anti-PLA2R antibodies (92 %, 49 of 53) than in anti-PLA2R antibody negative iMN patients (68 %, 32 of 47). Although no other significant differences were observed in patients with and without anti-PLA2R antibodies, anti-PLA2R antibody positive iMN patients had higher urinary protein levels (*p* = 0.06) and a higher rate of nephrotic syndrome (urinary protein ≥3.5 g/day and serum albumin ≤3 g/dL, *p* = 0.08) and a lower serum total protein level (*p* = 0.06) than the anti-PLA2R antibody negative iMN patients.

**Table 2:** Comparison of the patient’s characteristics between anti-PLA2R antibody positive and negative patients with idiopathic membranous nephropathy

Anti-PLA2R antibody	Negative (*n* = 47)	Positive (*n* = 53)	*P* value
Male, *n* (%)	31 (66)	32 (60)	0.56
Age at diagnosis (years)	68 ± 9	67 ± 9	0.48
Urinary protein (g/day)	3.7 (2.4–5.8)	4.6 (2.9–8.6)	0.06
Urinary protein ≥3.5 g/day, *n* (%)	24 (51)	35 (66)	0.13
Serum albumin (g/dL)	2.5 ± 0.9	2.3 ± 0.5	0.21
Serum albumin ≤3 g/dL, *n* (%)	32 (68)	49 (92)	0.02
Both urinary protein ≥3.5 g/day and serum albumin ≤3 g/dL, *n* (%)	21 (45)	33 (62)	0.08
Serum total protein (g/dL)	5.4 ± 0.9	5.1 ± 0.7	0.06
Serum creatinine (μM)	70.7 (61.0–91.9)	73.4 (61.9–86.2)	0.74
eGFR (ml/min/1.73 m^2^)	66.0 (50.0–78.0)	65.0 (56.0–76.5)	0.69
Serum IgG (mg/dL)	791 (594–1062)	668 (536–982)	0.33
Serum total cholesterol (mM)	7.5 (6.1–9.9)	7.6 (6.4–9.2)	0.90

The data are expressed as the number (%), mean ± SD or median (interquartile range). eGFR (mL/min/1.73 m^2^) = 194 × Serum creatinine (mg/dL)^−1.094^ × Age^−0.287^ × 0.739 (if female)

*Anti-PLA2R* anti-phospholipase A2 receptor autoantibody, *iMN* idiopathic membranous nephropathy, *eGFR* estimated glomerular filtration rate

**Fig. 3:**
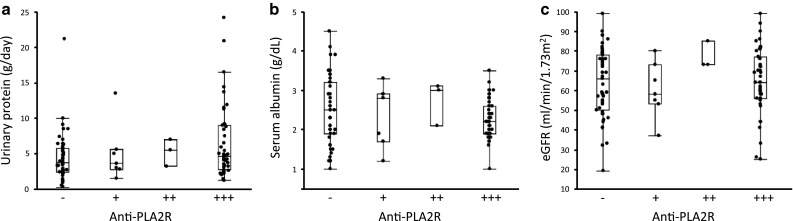
The relationship between the semi-quantitative values of anti-PLA2R antibodies and patient characteristics. No significant difference was observed between the levels of anti-PLA2R antibodies (−, negative; +, low; ++, middle; +++, high) and urinary protein (**a**), serum albumin (**b**), or eGFR (**c**)

We then examined the relationship between the semi-quantitative value of anti-PLA2R antibodies and patient characteristics. The levels of urinary protein, serum albumin, and eGFR were not different among iMN patients with negative, low, middle, and high titer levels (Fig. 3).

## Discussion

The present study revealed that the prevalence of anti-PLA2R antibodies is 53 % in Japanese patients with iMN. Although it was not significantly different, the prevalence of anti-PLA2R antibodies in iMN patients with nephrotic syndrome (61 %) was higher than that of iMN patients without nephrotic syndrome (61 % vs. 43 %, *p* = 0.08). The prevalence was lower than that found (70 %) in the original study by Beck et al. [7]. No patients with sMN were positive for anti-PLA2R antibodies.

The prevalence of anti-PLA2R antibody positive iMN patients from other countries is shown in Table 3. Previous studies have shown that the levels of urinary protein are significantly associated with the presence and titers of anti-PLA2R antibodies. Thus, the comparison of prevalence should be done among patients with nephrotic range proteinuria. The prevalence of anti-PLA2R antibodies in Chinese and Korean nephrotic patients was high, at 98 and 80 % [12, 13], respectively. In other cohorts, excluding one from Germany [14], the prevalence was 78–85 % in iMN patients with nephrotic range proteinuria (UP ≥3.5 g/day) [7, 8, 12, 13]. In contrast, 61 % of Japanese patients were positive for anti-PLA2R antibodies, even among iMN patients with nephrotic syndrome. The prevalence of anti-PLA2R antibodies in Japan was as low as that in the German cohort (66 %) [14]. The cohort from Korea showed a significant correlation between levels of urinary protein and serum albumin and the existence of circulating anti-PLA2R antibodies [12]. In our study, a low serum albumin was significantly associated with PLA2R positivity. No definitive conclusion can be made from our cohort, but the presence of anti-PLA2R antibodies may have induced more severe disease.

**Table 3:** Comparison of the prevalence of anti-PLA2R autoantibody in patients with iMN among different countries

Country	Method	Antigen	All patients	Patients with proteinuria ≥3.5 g/day	References
China	WB	rPLA2R	59/60 (98)	59/60 (98)	Qin et al. [13]
The Netherlands	WB	rPLA2R	14/18 (78)	14/18 (78)	Hofstra et al. [8]
Korea	WB	HGE	69/100 (69)	60/75 (80)	Oh et al. [12]
USA	WB	HGE	26/37 (70)	17/20 (85)	Beck et al. [7]
Germany	IIFT	rPLA2R	52/100 (52)	23/35 (66)	Hoxha et al. [14]
Japan	WB	HGE	53/100 (53)	33/54 (61)^a^	Present study

The data are expressed as the number of positive case/negative case (positive rate, %)

*WB* Western blot; *IIFT* indirect immunofluorescence test, *rPLA2R*, recombinant phospholipase A2 receptor, *HGE* human glomerular extract

^a^ Both urinary protein ≥3.5 g/day and serum albumin ≤3 g/dl

Why is there a low prevalence of anti-PLA2R antibody in Japanese patients with MN? There are several possible reasons for the discrepancy. First, there can be a genetic difference between people in Japan and other countries. However, it may be difficult to explain the difference in genetic background between Japanese, Korean, and Chinese cohorts. Second, Japan has a special health check-up system to detect low levels of proteinuria [16]. Japanese iMN patients may be diagnosed earlier than patients in other countries; therefore, the anti-PLA2R antibody levels might have been under the detection limit when they underwent renal biopsy. Third, unknown environmental or dietary factors may have affected the results. The factors causing the differences need to be clarified in future studies. Fourth, in Japanese iMN patients, other antigens may also play a role in the development of iMN. Studying Japanese patients with iMN may elucidate new pathogenic antigens. Lastly, technical differences may exist between countries. Although Western blot analysis with chemical luminescence is the most common technique for the detection of anti-PLA2R antibody, there are some problems with chemical luminescence for the detection of anti-PLA2R antibody in serum. For example, the bands of anti-PLA2R antibody were covered by high background signal if serum of low dilution was used, so a high dilution was necessary to prevent the high background. Bleached reactive bands often occurred due to enzymatic over reaction. The detection range of anti-PLA2R antibody concentration per image is narrow. Therefore, it is difficult to detect a low level of circulating anti-PLA2R antibody by chemical luminescence. On the other hand, the original detection method developed by us that employs an optimized chromogenic reaction with TMB resulted in a high resolution and low background without loss of sensitivity. In our method, the serum samples can be used without dilution, leading to a clear positive signal even in serum with a very low level of anti-PLA2R antibodies. In addition, our method is easy to use because it required only a digital camera or scanner for acquiring reactive band images, and a high-priced CCD imager is not needed. In the present study, we found that 7 iMN patients, who were negative at 1:10 dilution, became positive when examined without sample dilution. We also used the positive and negative control serum from Dr. D. Salant’s laboratory and confirmed that our system gave the same results as those in the original study. Therefore, it is unlikely that the low prevalence in our study is attributable to our detection system.

Western blot is considered the best method for qualitative measurement of anti-PLA2R antibody, whereas a quantitative method such as ELISA has considerable importance in the study of MN. Recently, several types of immunoassay systems for measuring circulating anti-PLA2R antibody have been developed, such as a commercial cell-based immunofluorescence assay (CBA-IIFT, Euroimmun AG), a commercial ELISA (Euroimmun AG), and an addressable laser bead immunoassay (ALBIA).The CBA-IIFT, like the Western blot, is used only for semi-quantitative measurements, but the ELISA and the ALBIA are quantitative assays. Behnert et al. reported that these three different immunoassays showed similar efficacies in the detection of anti-PLA2R antibodies in patients with iMN [17]. We are currently trying to evaluate the performance of CBA-IIFT and ELISA using serum from Japanese patients with iMN, sMN, and other renal diseases.

Our study has several limitations. We performed the Western blot analysis using HGE, not PLA2R protein, as an antigen. It is possible that we detected other proteins with similar molecular weight at around 180 kDa. If this is the case, the real prevalence of anti-PLA2R antibody may be even lower. We studied patients only in the Tokai area, 1 limited region of Japan, and our findings may not be generalizable to iMN patients in other areas of Japan. Future studies using recombinant PLA2R as an antigen and enrolling patients in other regions of Japan will clarify these problems.

In conclusion, we determined that the prevalence of anti-PLA2R antibodies in Japanese patients with iMN is approximately 50 %, which was similar to previous reports from Germany but lower than reports from Asian countries (China and Korea). This may indicate that the presence of other pathogenic antigens plays a significant role in Japanese patients with iMN. Anti-PLA2R antibodies were not found in any patients with sMN.
